# Fabrication of Printable Colorimetric Food Sensor Based on Hydrogel for Low-Concentration Detection of Ammonia

**DOI:** 10.3390/bios13010018

**Published:** 2022-12-23

**Authors:** Mirim Ham, Soohyun Kim, Wonmok Lee, Hyunjung Lee

**Affiliations:** 1 School of Materials Science and Engineering, Kookmin University, 77 Jeongneung-ro, Seongbuk-gu, Seoul 02707, Republic of Korea; 2 School of Materials Science and Engineering, Yeungnam University, 280 Daehak-ro, Bukbu-dong, Gyeongsan-si 38541, Republic of Korea; 3 Department of Chemistry, Sejong University, 98 Gunja-ro, Gwangjin-gu, Seoul 143747, Republic of Korea

**Keywords:** hydrogel, colorimetric sensor, food sensor, printable sensor, ammonia, PAN

## Abstract

With the increasing market share of ready-to-cook foods, accurate determination of the food freshness and thus food safety has emerged as a concern. To commercialize and popularize food sensing technologies, food sensors with diverse functionalities, low cost, and facile use must be developed. This paper proposes printable sensors based on a hydrogel-containing pH indicator to detect ammonia gas. The sensors were composed of biocompatible polymers such as 2-hydroxyethyl methacrylate (HEMA) and [2-(methacryloyloxy)ethyl] trimethylammonium chloride (MAETC). The p(HEMA-MAETC) hydrogel sensor with bromothymol blue (BTB) demonstrated visible color change as a function of ammonia concentration during food spoilage. Furthermore, polyacrylonitrile (PAN) was added to improve transport speed of ammonium ions as the matrix in the sensors and optimized the viscosity to enable successful printing. The color changed within 3 min at ammonia concentration of 300 ppb and 1 ppm, respectively. The sensor exhibited reproducibility over 10 cycles and selective exposure to various gases generated during the food spoilage process. In an experiment involving pork spoilage, the color change was significant before and after exposure to ammonia gas within 8 h in ambient conditions. The proposed sensor can be integrated in bar codes and QR codes that are easily mass produced.

## 1. Introduction

With the increasing number of single-person households and social distancing requirements for preventing COVID-19 transmission, the demand for convenience foods, instant foods, and delivery foods is rising. Foods without an expiration date are frequently disposed of owing to lack of information regarding their quality and spoilage. The food safety is also threatened by the lack of accurate indicators for the food freshness. To address these problems, food sensors such as biosensors [1,2,3,4] and gas sensors [5,6,7,8] have been developed, depending on the target materials to be detected and their receptors [9,10,11,12]. Conventional sensors are typically based on electronic devices that are expensive and difficult to use. It is desirable for consumers to be able to determine the freshness of food and degree of spoilage intuitively and simply. For example, fish generates gases such as trimethylamine (TMA), total volatile basic nitrogen, sulfur compounds, carbon dioxide, aldehyde, ketones, and esters [13,14,15,16]. Because the concentration of nitrogen compounds (ammonia, TMA) increases as food spoils, they must be promptly detected during storage to identify food decomposition [17]. Therefore, many researchers have used colorimetric methods to identify food spoilage visually and instantly [14,18,19,20].

Specifically, colorimetric gas sensors based on 10,12-pentacosadiynoic (PCDA) were prepared for detecting ammonia [18,19,20,21]. Ammonia reacts with the -COOH group of PCDA to produce carboxylate anions (COO^−^) and ammonium cations (NH_4_^+^). The repulsive forces between the COO^−^ groups rearrange the PCDA chains, leading to color variations. The reproducibility and stability of this color change are high. However, PCDA is expensive, and the sensor fabrication process is complex. In addition, because the color before exposure to ammonia may be influenced by UV irradiation, the initial color may change under prolonged exposure to light, rendering it challenging to accurately identify the freshness of food.

Colorimetric sensors with pH indicators are a low cost, simple method to detect gases in acidic or basic environments. [11,22,23,24,25]. To show more information, array-based sensors are fabricated containing various range of pH indicator. It is easier to save and monitor food freshness by connecting with consumers’ mobile devices [26,27,28,29,30]. Moreover, such sensors can be easily attached to food wrappers to commercialize and popularize food monitoring technologies. However, the pH indicator is generally fixed to a matrix (e.g., membrane, sol-gel), which detects in high gas concentration (>1 ppm) scenarios due to the acidic preparation environment or the lack of materials that dissociate pH indicators [31]. Additionally, sensors cannot be transformed into various structural forms. 

To address these problems, we prepared a printable sensor based on a hydrogel-containing pH indicator. Owing to its compatibility with printing technologies. the proposed sensor can be produced in a text or barcode form, and the degree of food spoilage can be identified through connectivity with a mobile device [26,32]. To ensure printability, polyacrylonitrile (PAN) was incorporated in the hydrogel as the viscous agent and a matrix to enable the rapid transport of ions [23]. The key parameters for a colorimetric sensor are its detection sensitivity and reproducibility of color change. The fabricated sensor could detect ammonia at low concentrations because the water inside the hydrogel dissociated ammonia (NH_3_ + H_2_O → NH_4_^+^ + OH^−^). To enhance the sensor sensitivity, the degree of color change and diffusion rate were optimized by varying the compositions of hydrogel materials. In general, a positively charged functional group of trimethylammonium (−N^+^(CH_3_)_3_) of monomer consisting of hydrogel electrostatically interacts with the negatively charged sulfonate group (SO_3_^−^) of the pH indicator and thus exhibits excellent color reproducibility and stability [33,34]. Consequently, the proposed sensor could enable fast detection. Overall, the proposed printable sensor can be easily manufactured and will help to visual understand the state of food in a facile manner.

## 2. Materials and Methods

### 2.1. Materials

2-Hydroxyethyl methacrylate (HEMA, 97%, Sigma–Aldrich, St. Louis, MO, USA) and [2-(methacryloyloxy)ethyl] trimethylammonium chloride (MAETC, 80 wt% in H_2_O, Sigma–Aldrich, St. Louis, MO, USA) were used as co-monomer scaffolds to prepare the hydrogel sensor. Ethylene glycol dimethacrylate (EGDMA, 98%, Sigma–Aldrich) and 2,2-dimethoxy-2-phenylacetophenone (DMPA, trade name-IRGACURE 651, BASF, Floham Park, NJ, USA) were used as the cross-linking agent and photo-initiator, respectively. PAN (Mw = 150,000, Sigma–Aldrich, St. Louis, MO, USA) was applied as a viscosity control agent and ion transport matrix. Bromothymol blue (BTB, Sigma–Aldrich, pH 6.0: yellow, pH 7.6: blue) was used as the pH indicator. Dimethyl sulfoxide (DMSO), ammonium hydroxide solution, acetic acid, chloroform, and ethyl alcohol, and pH 4/11 buffer solutions were purchased from Daejung (Siheung, Gyonggi, Korea) and used as received.

### 2.2. Fabrication of p(HEMA-MAETC) Based Hydrogel Sensor

**Figure 1:**
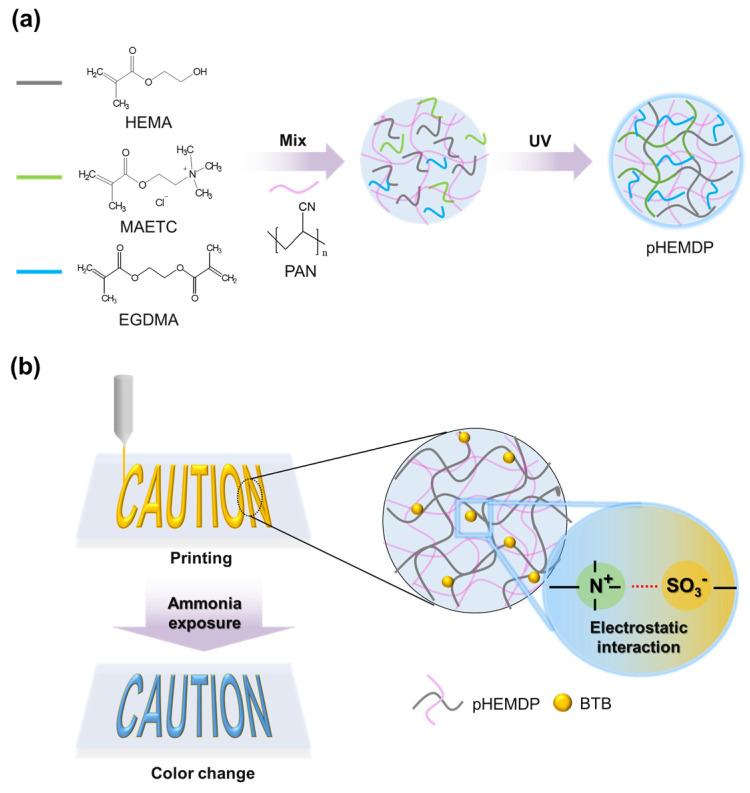
Schematic of (**a**) photopolymerization mechanism and (**b**) preparation of pHEMDP hydrogel sensor.

To prepare the printable p(HEMA-MAETC) with DMSO and PAN (pHEMDP) hydrogel, 40 wt% of MAETC monomers were mixed with HEMA solution. In general, a higher composition of MAETC in p(HEMA-MAETC) is associated with a higher absorption of water owing to (−N^+^(CH_3_)_3_) groups in MAETC; however, its mechanical properties deteriorate [35]. PAN was used to increase the viscosity of the solution [36] through increased hydrogen bonding between PAN and the solvent molecules [37]. The 6 wt% of PAN was slowly added to monomer solution, which has been noted to yield an adequate viscosity in printing applications (Figure S1 in Supplementary Materials). The printing process schematic is shown in Figure 1b. To increase the solubility of PAN, DMSO which is a good solvent for PAN was used as co-solvent with deionized water (DI water), a ratio was DMSO:DI water = 40:60 (mol%). The weight fraction of EGDMA and DMPA was 1 wt% and 1.5 wt% to the monomer solution (HEMA and MAETC), respectively. The BTB was added 4mM to a prepared solution. For comparison, we prepared p(HEMA-MAETC) (pHEM) and p(HEMA-MAETC) with DMSO (pHEMD) hydrogels to evaluate the characteristics of the pHEMDP hydrogel sensors. The fabrication method was identical, with the following exceptions: In the former and latter cases, DI water and a mixture of DI water and DMSO were used as the solvent, respectively, and PAN was not added. Detailed components and ratios of each hydrogel sensor are indicated in Table 1.

Prepared solution was printed by doctor-blade method at 1 cm/s rate and dimension of 1 cm × 1 cm and height of 0.1 cm on a polytetrafluoroethylene (PTFE) substrate, and then the sensors were photopolymerized in a UV oven (RX-CB400, Carima, Seoul, Korea) for 10 min. 

### 2.3. Characterization

Photopolymerized hydrogels were prepared as pH sensors after swelling in DI water. To check the amount of water contained in the hydrogel, The water content was calculated using Equation (1):

(1) $$Water\;content\;\%=\left(W_{s}-W_{d}\right)/W_{d}\times 100,$$

where *W_s_* and *W_d_* are the mass values of the swelled hydrogel for a certain time and dried hydrogel, respectively. Freeze drying method is a low temperature dehydration process and is one of the common methods used to prepare fully dried samples. We prepared dried hydrogels by freeze-dry method to completely remove the water in hydrogels for 24 h and compared their weight before and after freeze-drying. 

To evaluate the sensing properties of the printed hydrogel sensors, the samples (1 cm × 1 cm) were exposed to ammonia (vapor concentration ranging from 100 ppb to 1 ppm) derived from different volumes of ammonium hydroxide solution. The vapor concentrations were calculated based on the ideal gas equation [18]. The samples were also exposed to 1 ppm of acetic acid, chloroform, and ethyl alcohol in a closed system [11,18]. The reflectance in each trial was measured at 581 nm using a spectrometer (AvaSpec-3648, Avantes, Apeldoorn, The Netherlands) with an optical microscope (S39A, Le.am solution, Siheung, Gyonggi, Republic of Korea). Specifically, the intensity of color change after exposure to ammonia gas was expressed as the RGB distance from the origin before ammonia exposure in color coordinates, using Equation (2) [23,38]:

(2) $$RGB\;distance=\sqrt{{\left(R_{b}-R_{a}\right)}^{2}+{\left(G_{b}-G_{a}\right)}^{2}+{\left(B_{b}-B_{a}\right)}^{2}},$$

where $R_{x}$,$\;G_{x}$, and $B_{x}$ represent the average values of red, green, and blue, respectively, and a and b represent the color of the reference and sample, respectively. Each RGB value was extracted from an optical image. RGB distance is a useful guide for the qualitative investigation by naked eyes when visual color changes occur over time. The investigation based on RGB distance is advantageous in that it can detect multiple dye spots at once and is easy to compare overall. All experiments were conducted in closed system.

To examine the reproducibility of the color change with pH variations, pHEMDP was alternately immersed in the pH 4 and pH 10 buffer solutions, and the reflectance was measured after complete color change in each pH solution, for over 10 cycles. 

### 2.4. Evaluation of Sensing Characteristics for Food Spoilage

To see the possibility of our sensors as food sensors, pHEMDP was printed on label paper using a syringe with 26 G nozzles at constant speed at 1 mL/h through a syringe pump to evaluate its ability of food spoilage detection. After photo-curing, the printed pHEMDP was attached to a plastic container containing 150 g pork. The food container with pHEMDP was stored in a refrigerator at 2 °C and in ambient conditions (temperature (20–25 °C) and humidity (20–23%)) to compare the different phenomenon of food spoilage and observed over time. 

## 3. Results

### 3.1. Optimization of Sensing Properties of Hydrogel Based Sensor

**Figure 2:**
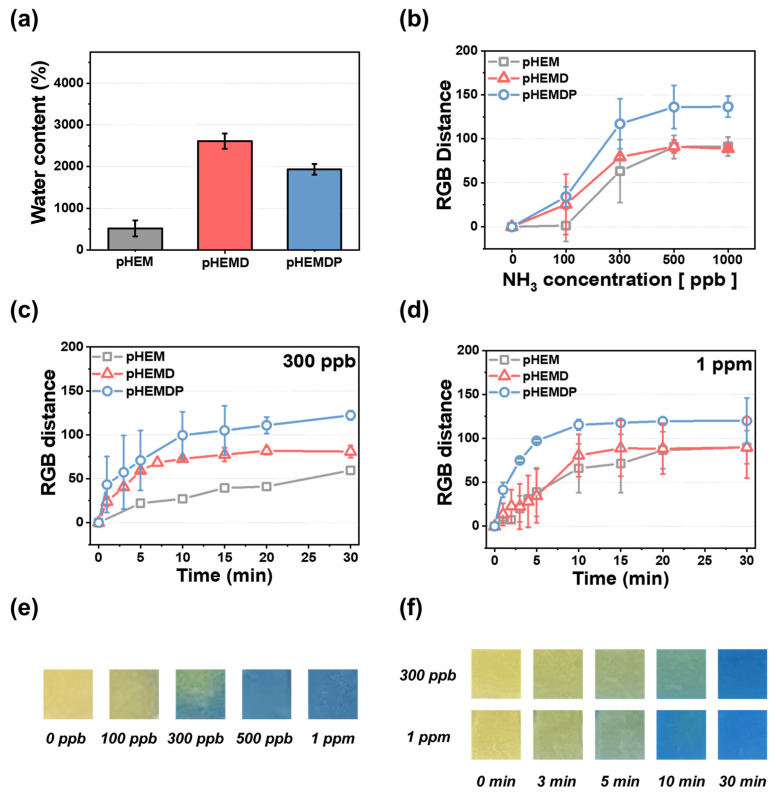
(**a**) Water contents of different types of hydrogels and (**b**) calibration curve of RGB distance and different NH_3_ concentrations after 30 min: addition of DMSO and PAN hydrogels. Rate at ammonia concentrations of (**c**) 300 ppb and (**d**) 1 ppm in 30 min. The standard deviation was obtained through 5 samples for each hydrogel. Optical images of pHEMDP hydrogel (**e**) at different NH_3_ vapor concentrations (0–1 ppm) and (**f**) as a function of detection time after NH_3_ vapor exposure at 300 ppb and 1 ppm.

To determine the characteristics of color change as a function of each component of the hydrogel sensor, the water contents of hydrogels consisting of different materials were compared (Figure 2a). The photopolymerized hydrogels were allowed to swell for more than two hours in DI water and then used as sensors. (Figure S2). The color of the sensor changed owing to change in pH resulting from the diffusion of ammonia gas that dissociated by water inside the hydrogel. The pHEM hydrogel had a water content of approximately 518%. When DMSO was introduced, the water content increased significantly to 2612% owing to the hydrogen bonding between the DMSO and water molecules [37]. 

Humans can smell the gases generated during food spoilage when their concentrations are approximately 35 ppm or higher [39]. Therefore, food sensors must be able to detect the target material at low concentrations. The dependance of the color change on the ammonia concentration was examined by exposing the sensor to ammonia (concentrations of 100–1000 ppb) for 30 min and comparing the sensitivity of hydrogel materials with different water content (Figure 2b). pHEM exhibited only a slight color change until 100 ppb. However, the RGB distance began to increase at approximately 300 ppb, with the RGB distances at 500 ppb and 1 ppm being 90. In contrast, pHEMD and pHEMDP changed color with an RGB distance of 25 or higher at 100 ppb concentrations. At higher concentrations of ammonia, pHEMDP exhibited a higher RGB distance (=130) than pHEMD (RGB distance = 90). In comparison of pHEM and PHEMD, there is no difference of RGB distance at high concentration (>500 ppb). However, RGB distance of pHEMD was higher than pHEM at low concentration (<300 ppb), which attributed to water content of hydrogel. Figure 2e shows the optical image of the color change of pHEMDP. The sensor turned from yellow to dark green and blue at 100 ppb ammonia concentrations. The color completely changed at concentrations higher than 300 ppb. The RGB distance values significantly increased in the range of 100 ppb to 300 ppb. In other words, the pHEMDP hydrogel sensor could change color even when exposed to trace concentrations of ammonia (below 300 ppb).

The time dependence of color change is a key property of colorimetric sensors. Therefore, we compared the change in the color of sensors consisting of different materials as a function of the sensing time when subjected to 300 ppb and 1 ppm ammonia concentrations. Figure 2c,d shows the results for the optimized sensors exposure to ammonia at various instances in a period of 30 min. The slope of the graph indicates the rate of color change. The RGB distance of pHEM reached saturation after 30 min for both concentrations. In comparison of pHEMD and pHEMDP, pHEMDP reaches the same RGB distance faster than pHEMD when exposed to 300 ppb and 1 ppm ammonia. The DMSO makes the polarity of PAN weaken and forms the solvent bridge when PAN incorporate with DMSO, leading to enhance the rate of color change by facilitating the ammonium (NH_4_^+^) ions and hydroxide ion (OH^−^) [35,40,41,42]. The corresponding optical images are shown in Figure 2f. At 300 ppb, the color changed from yellow to green under 3 min and then to blue after 15 min. In contrast, at 1 ppm, the color rapidly changed from yellow to blue within 10 min.

### 3.2. Characterization of pHEMDP

**Figure 3:**
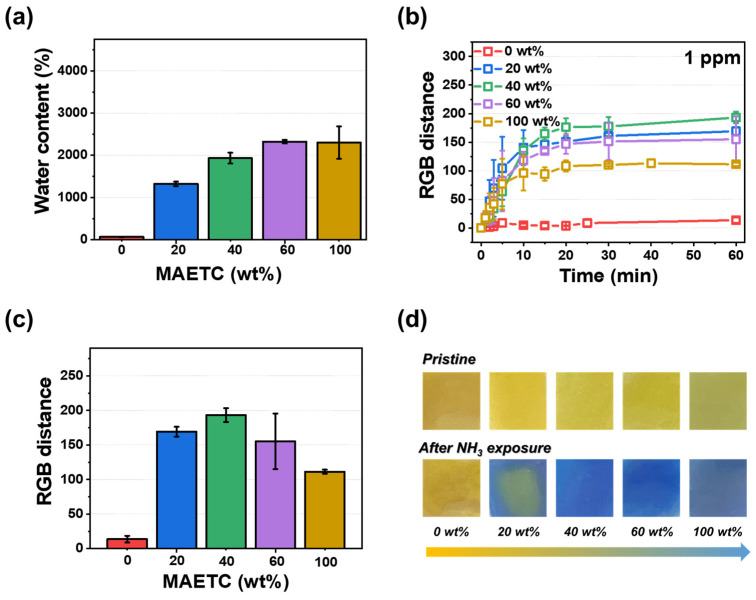
Effect of MAETC content in pHEMDP sensor: (**a**) water content and (**b**) calibration curve of RGB distance and time at 1 ppm vapor concentration of ammonia. The standard deviation was obtained through 5 samples for each hydrogel. Corresponding (**c**) RGB distance and (**d**) optical images after 60 min.

The sensing performance of pHEMDP was optimized by varying the ratio of the co-monomer. Figure S3 shows the performance as a function of the water content and time. pHEMDP reached equilibrium swelling after 2 h. Figure 3a shows the water content after 24 h of swelling with different MAETC contents. As the MAETC content increased, the content of -N(CH_3_)_3_^+^ increased, resulted in increased water content [34]. To evaluate the sensing performance, the RGB distance of pHEMDP at different times were compared. The sensor was exposed to 1 ppm ammonia, and the measurements were obtained after reaching equilibrium swelling (Figure 3b,c). Although the detection time was independent of the water content, the RGB distance was the highest at 40 wt% of MAETC. The water influenced the RGB distance. In the case of MAETC 0 wt%, there was no color change because of rarely contained water, and leakage of BTB occurred during swelling owing to the absence of ammonium groups that can interact with BTB (Figures S4 and S5). With the increase in the MAETC content, the amount of ammonia dissociated by water molecules increased, thereby intensifying the pH change inside the hydrogel. However, the RGB distance decreased as the MAETC content increased to more than 60 wt%. Because pHEMDP initially appeared slightly green before exposure to ammonia. Figure 3d shows the optical image of the sensor with different MAETC contents before and after exposure to ammonia.

**Figure 4:**
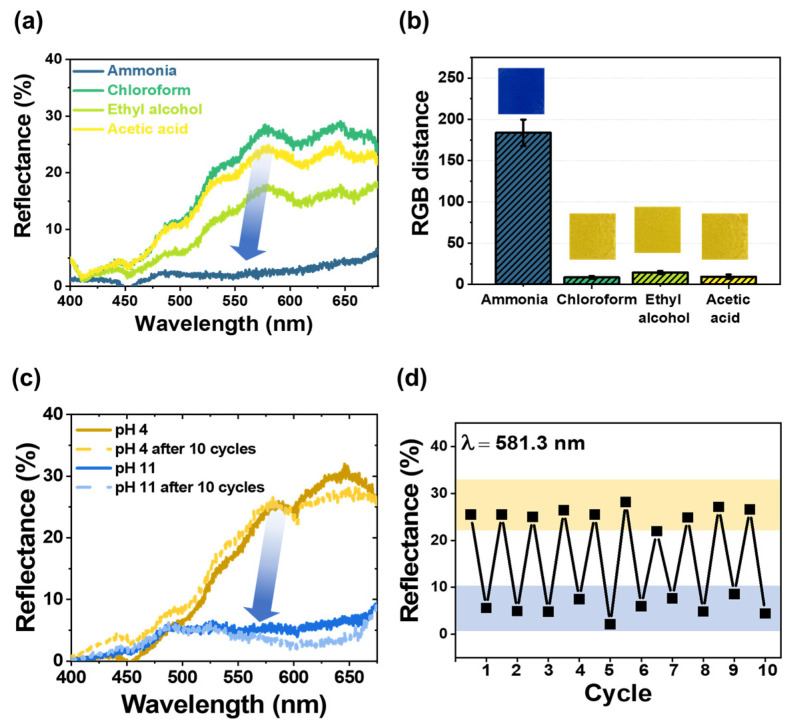
(**a**) Reflectance spectra and (**b**) bar plot showing the RGB distance and optical images of pHEMDP exposed to various gases with a concentration of 1 ppm for 30 min. The standard deviation was obtained through 5 samples for each hydrogel. (**c**) Reflectance spectra before and after 10 cycles, (**d**) reflectance after exposure to pH 4 and 11 solutions.

To evaluate the sensor’s selectivity toward ammonia, the changes in the color were compared with those occurring during exposure to ammonium hydroxide solution, acetic acid, ethyl alcohol, and chloroform [18,22,23,43], which are generated during food spoilage. The results are shown in Figure 4a,b in terms of the reflectance and RGB distance values, respectively. In the reflectance–wavelength graph, the reflectance disappeared in the yellow wavelength region (λ = 565 to 590 nm) in the sensors exposed to ammonia. Similarly, the RGB distance when the sensor was exposed to ammonia (=180) was larger than that when it was exposed to other gases (<15).

Furthermore, the reproducibility of pHEMDP was tested. Reflectance spectra were obtained by dipping the sensor 10 times alternately in an acidic (pH = 4) and basic (pH = 11) solution after it completely changed color (Figure 4c). In the case of the acidic solution, a strong and broad reflection band was observed in the yellow light region. In the case of the basic solution, the reflection band in the yellow light region disappeared, and the remaining reflection band was observed in the blue light region (λ = 440 to 485 nm). Figure 4d shows the reflectance intensity at 581.3 nm recovered in 10 cycles of alternate dipping in the acidic and basic solutions. The reflectance at pH 4 and 11 was approximately 30% and less than 10%, respectively. Based on this reversible behavior of pHEMDP, we concluded that the ionic bonding between the ammonium cation of MAETC in the hydrogel and anionic sulfonate group of the pH indicator was maintained.

### 3.3. Use of the Hydrogel Sensor for Detecting Food Spoilage

**Figure 5:**
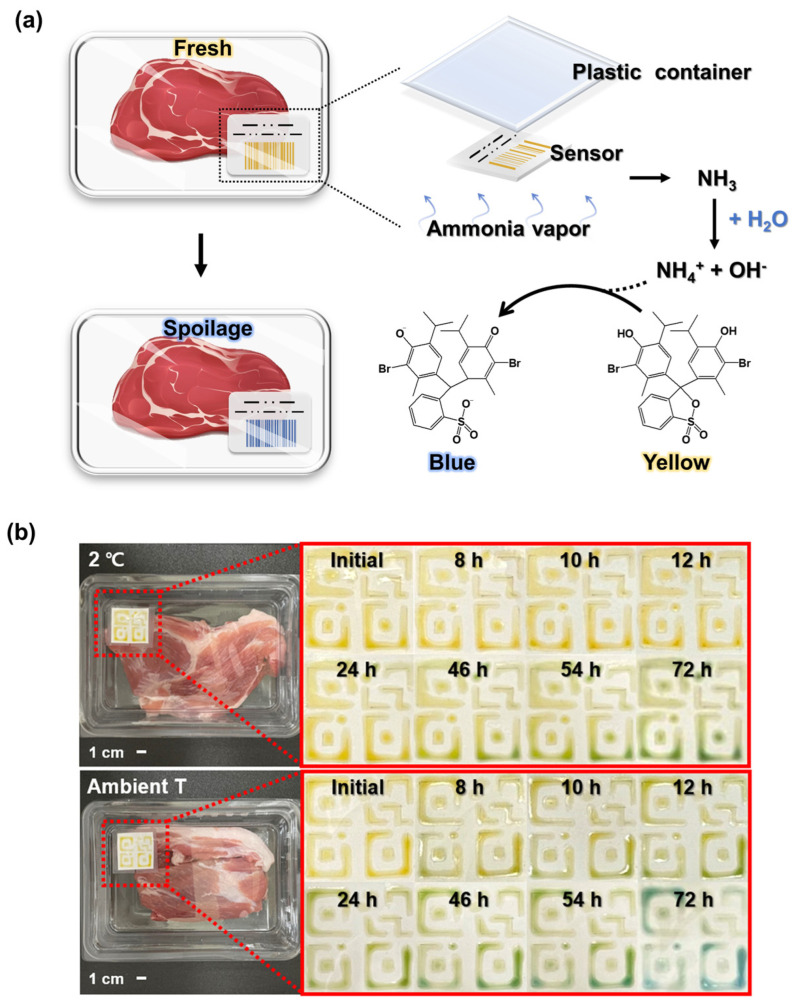
(**a**) Schematic of sensor structure. (**b**) Optical images of sensor application to 150 g of pork and comparison of color changes at different times in a refrigerator and under ambient temperature.

We printed the optimized pHEMDP sensors and evaluated their performance in actual food sensing scenarios. A patch was attached to a conventional food container containing pork (Figure 5a). The food container with the sensor was stored in the refrigerator and at an ambient temperature, and the change in the sensor color was compared. The pork stored at ambient temperature spoiled faster than the pork stored in the refrigerator, resulting in a rapid change in the color of the pHEMDP sensor. After 8 h, the sensor in the ambient temperature condition began exhibiting a green color that darkened and then became blue in 72 h, indicating spoilage. These findings highlighted the potential of the pHEMDP sensor as a food sensor.

## 4. Conclusions

We prepared a food sensor that could provide visual indications of food spoilage through changes in the color of the pH indicator when exposed to ammonia. The sensor performance was evaluated by comparing three types of hydrogels: pHEM, pHEMD, and pHEMDP. pHEMDP exhibited the largest RGB distance and lowest detection time at all ammonia concentrations. The performance was optimized by controlling the monomer ratio. pHEMDP consisting of 40 wt% MAETC exhibited the largest RGB distance and fast-sensing property. The color changed within 3 min when exposed to 300 ppb and 1 ppm ammonia. In addition, pHEMDP was selected toward ammonia among the gases typically produced during food spoilage. Its reproducibility was demonstrated by comparing the reflectance when the sensor was alternately dipped into pH 4 and 11 solutions for 10 cycles. Finally, the pHEMDP sensor was printed and attached to a container containing real food. The color changed within 8 h in ambient condition. The proposed printable pHEMDP can be integrated into barcodes and QR codes and commercialized owing to its ability to be mass produced in a low-cost manner. The proposed framework is thus a promising alternative as an attachable, reusable, and easy-to-use sensor for smart food packaging.

## Figures and Tables

**Table 1:** The type of monomer and solvent in a different component of hydrogel sensors.

	Monomer	Solvent
pHEM	HEMA:MAETC = 1:0.8 (*w*/*w*)	DI water
pHEMD	HEMA:MAETC = 1:0.8 (*w*/*w*)	DI water, DMSO
pHEMDP	(HEMA:MAETC):PAN = 1:0.06 (*w*/*w*)	DI water, DMSO

## Data Availability

Not applicable.

## Supplementary Materials

The following supporting information can be downloaded at: https://www.mdpi.com/article/10.3390/bios13010018/s1, Figure S1: Optical image of hydrogel solution for maintenance of their pattern depending on the amount of PAN after directly printing on the polyethylene terephthalate (PET) substrate using syringe at constant speed at 1 mL/h through a syringe pump; Figure S2: Water content of pHEM, pHEMD, and pHEMDP hydrogel as a function of swelling time in DI water; Figure S3: Water content of pHEMDP by different weight of MAETC in DI water for 24 h; Figure S4: Optical image of BTB leaked from pHEMDP with different MAETC wt% in DI water after 4 h; Figure S5: The absorbance of BTB leaked from pHEMDP with different MAETC wt% in DI water after 4 h.
