# Supplementary material for: Fabrication of Printable Colorimetric Food Sensor Based on Hydrogel for Low-Concentration Detection of Ammonia
Source: Biosensors (Basel). 2022 Dec 23;13(1):18. doi: 10.3390/bios13010018 (PMC9856113; doi:10.3390/bios13010018)
Supplement: Supplementary file 1 [file biosensors-13-00018-s001.zip › biosensors-2086797-supplementary.pdf]

biosensors

Supplementary Materials

Fabrication of Printable Colorimetric Food Sensor Based on Hydrogel for Low-Concentration Detection

Mirim Ham, Soohyun Kim, Wonmok Lee and Hyunjung Lee

PAN (wt %)	1	2	3	4	5	6
Optical image	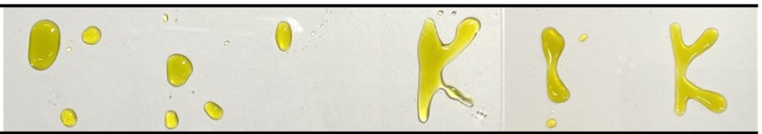					

Figure S1. Optical image of hydrogel solution for maintenance of their pattern depending on the amount of PAN after directly printing on the polyethylene terephthalate (PET) substrate using syringe at constant speed at 1 ml/h through a syringe pump.

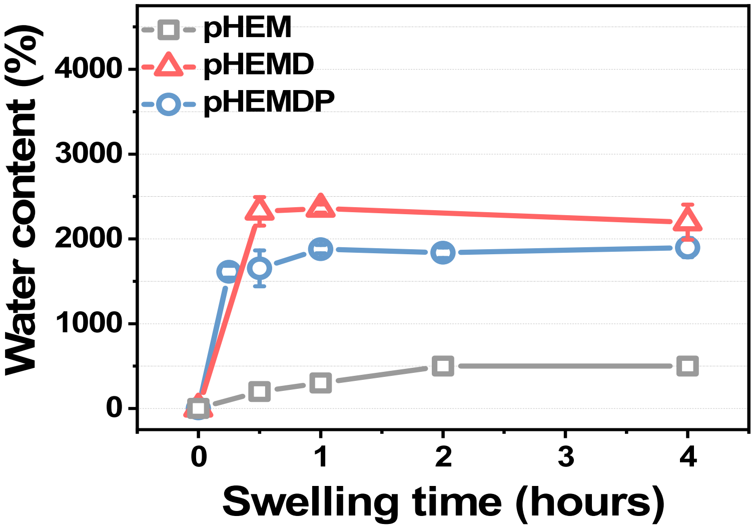

Figure S2. Water content of pHEM, pHEMD, and pHEMDP hydrogel as a function of swelling time in DI water.

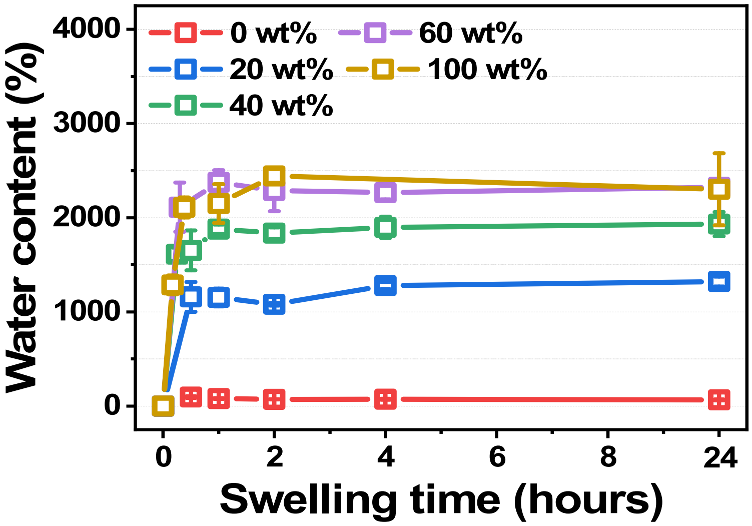

Figure S3. Water content of pHEMDP by different weight of MAETC in DI water for 24 h.

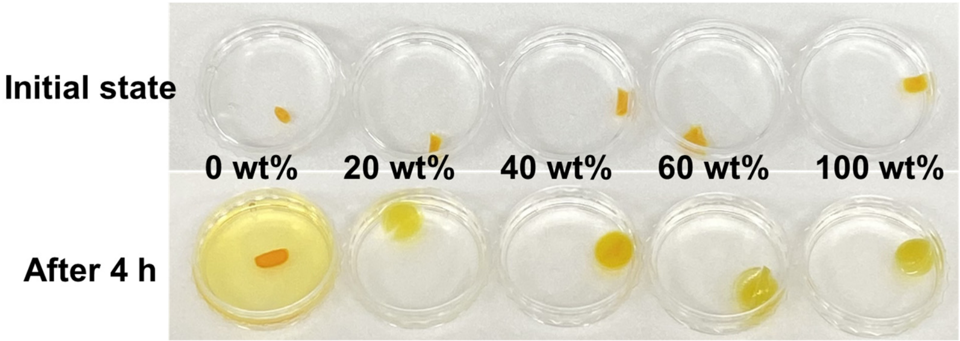

Figure S4. Optical image of BTB leaked from pHEMDP with different MAETC wt% in DI water after 4 h.

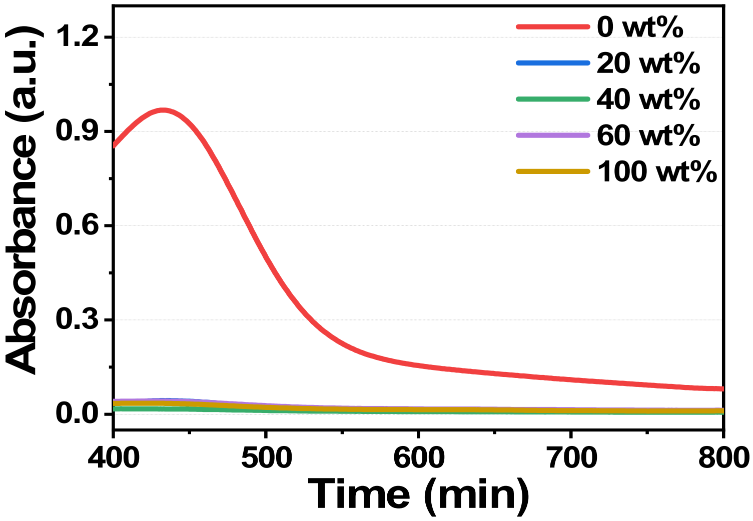

Figure S5. The absorbance of BTB leaked from pHEMDP with different MAETC wt% in DI water after 4 h.
